# Outcome markers of ART-treated HIV+ patients with early stage Kaposi’s sarcoma

**DOI:** 10.1371/journal.pone.0235865

**Published:** 2020-07-07

**Authors:** Owen Ngalamika, For Yue Tso, Salum Lidenge, Sody Munsaka, Danielle Shea, Charles Wood, John West

**Affiliations:** 1 Dermatology & Venereology Section, University Teaching Hospitals, University of Zambia School of Medicine, Lusaka, Zambia; 2 School of Biological Sciences, University of Nebraska-Lincoln, Lincoln, Nebraska, United States of America; 3 Nebraska Center for Virology, Lincoln, Nebraska, United States of America; 4 Ocean Road Cancer Institute, Dar es Salaam, Tanzania; 5 Muhimbili University of Health and Allied Sciences, Dar es Salaam, Tanzania; 6 School of Biological Sciences, University of Zambia, Lusaka, Zambia; 7 Department of Biochemistry, University of Nebraska-Lincoln, Lincoln, Nebraska, United States of America; Penn State University School of Medicine, UNITED STATES

## Abstract

HIV-associated/epidemic Kaposi’s sarcoma (EpKS) is an AIDS-defining angio-proliferative malignancy. It can be treated with antiretroviral therapy (ART) alone or with ART plus cytotoxic chemotherapy. ART-treated EpKS can either respond or worsen upon treatment. This study aimed at identifying immunological markers of ART-treatment response. We compared responders (those with clinical EpKS tumor regression) versus poor responders (those with progressive or non-responsive EpKS). We measured plasma cytokine and chemokine levels using cytometric bead assays. Kaposi’s sarcoma herpesvirus (KSHV) neutralizing antibody (nAb) responses were also quantified to test associations with treatment outcome. Interleukin (IL)-5 levels were significantly elevated in responders versus poor-responders at baseline (0.76pg/ml *vs*. 0.37pg/ml; p<0.01) and follow-up (0.56pg/ml *vs*. 0.37pg/ml; p<0.01); IL-6 was lower in responders than poor-responders at follow-up (600fg/ml *vs*. 4272fg/ml; p<0.05). IP-10/CxCL-10 was significantly lower at follow-up in responders versus poor-responders (187pg/ml *vs*. 528pg/ml; p<0.01). KSHV nAb were not significantly differential between responders and poor-responders. In conclusion, high plasma IL-5 at baseline could be a marker for ART-treated KS tumor regression, whereas increased pro-inflammatory cytokine IL-6, and the chemokine IP-10, associate with KS tumor progression.

## 1. Introduction

Kaposi’s sarcoma (KS) is a vascular malignancy highly prevalent in sub-Saharan Africa (SSA) [1–3]. Human herpesvirus type 8 (HHV-8), which is also known as Kaposi’s sarcoma-associated herpesvirus (KSHV), has been implicated as the etiological agent of all four types of KS [4]. These are: i) Classic KS—occurs in elderly men of Mediterranean origin; ii) Iatrogenic KS—a result of immunosuppressive therapy; iii) Endemic KS—seen among the HIV-negative population in SSA; and iv) Epidemic or AIDS-associated KS (EpKS)–associated with HIV-induced immunosuppression. EpKS is the most common type of KS in SSA countries, and is an HIV stage 4 disease according to the World Health Organization. Despite the introduction of ART, the incidence, prevalence, and mortality attributed to EpKS remain high in SSA despite more substantial reductions in higher income settings like North America and Europe [2, 5].

It is clear from the epidemiological association of KS with HIV-induced immunosuppression, immunosuppressive therapy, and aging [6–8] that immune dysregulation plays a key role in the pathogenesis of KS. However, the fundamental mechanisms underlying KS development are largely unknown. Prior to the introduction of ART, EpKS was considered an AIDS-defining malignancy because it commonly developed when HIV viral loads were high and CD4 counts were very low. However, EpKS incidence levels have remained steady despite effective roll-out and uptake of ART throughout SSA. The majority of EpKS cases are now presenting after HIV viral suppression and at least partial immune reconstitution [9]. In addition, the recurrence rates after KS treatment are high [10].

Management of EpKS can be challenging due to difficulties in disease staging, adverse effects of available treatments, and poor outcomes. Early-diagnosed skin-limited EpKS is usually treated with ART alone, whereas advanced EpKS requires ART plus cytotoxic chemotherapy. Treatment of EpKS with ART results in variable outcomes including remission, stable disease, or progression requiring addition of cytotoxic cancer chemotherapy. Furthermore, EpKS patients initiating ART have increased risk of mortality when compared with non-KS HIV-infected individuals initiating ART [11]. Cytotoxic cancer chemotherapeutics can lead to bone marrow suppression resulting in anemia and leukocytopenia, and may also cause other adverse effects such as peripheral neuropathy, lung fibrosis, and cardiotoxicity in individuals who are already immunocompromised [12]. Factors associated with disease progression or remission as a result of ART are unknown. It is important to identify these factors so that chemotherapy can be avoided when possible, and individuals with early EpKS who will require both ART and chemotherapy can be identified early and treated appropriately.

Cytokines have been observed to be dysregulated in KS patients [13–15]. Anti-KSHV neutralizing antibodies (nAb) are known to be more readily detected in KS patients than in asymptomatic KSHV-infected controls [16], but it is not known how or whether nAb levels change in association with ART or any other KS treatment. Here, we quantified plasma cytokines and nAb in chemotherapy-naïve, early-diagnosed ART-treated KS patients to investigate their potential as prognostic biomarkers of disease progression or remission.

## 2. Materials and methods

### 2.1 Patients and samples

This study was conducted on adult KS patients presenting at the University Teaching Hospital (UTH) in Lusaka, Zambia. We recruited histologically-confirmed, early-stage (AIDS Clinical Trials Group Stage T0/S0) HIV-associated KS patients who were antiretroviral therapy (ART)-naïve or on ART for less than 2 weeks. Recruitment was done after obtaining informed consent. All recruited patients were 18 years and older, had a limited number of early KS lesions (patches, plaques, or nodules), lacked lymphedema, and had no evident visceral involvement. At baseline, sociodemographic information was collected, a physical assessment was conducted, and blood samples were collected. ART was initiated in those who were ART-naïve. All recruited patients were followed up monthly for at least 6 months for either remission (responders) or progression of KS (poor responders). Responders all had a follow up period of at least 6 months while poor responders had variable follow up ranging from 1 to 6 months depending on when they experienced disease progression. None of our study participants had co-infections such as malaria or hepatitis, or HIV-associated comorbidities such as tuberculosis or cryptococcal meningitis at baseline or during follow up. Venous whole blood was collected at baseline and at the time of progression or after 6 months for those who responded to ART. All blood samples were collected in EDTA vacutainers. Plasma was separated and then stored at -80°C until analysis. The study was approved by the University of Zambia Biomedical Research Ethics Committee, the Zambian National Health Research Authority, and the Institutional Review Board at the University of Nebraska-Lincoln.

### 2.2 HIV testing and viral load

HIV-1 status for all patients was determined as recommended by the Zambian Ministry of Health. The Alere Determine HIV-1/2 kit (Alere Medical Co. Ltd) was used for screening, while the SD Bioline HIV-1/2 kit (Standard Diagnostics Inc) was used for confirmation. HIV-1 plasma viral load was measured on the Hologic Panther (Hologic) using the Aptima HIV-1 Quant Dx Assay kit (Hologic) according to the manufacturer’s protocol.

### 2.3 CD4 T cell quantification

CD4 counts were determined with BD FACSCalibur (BD Biosciences) using the BD TriTest kit (BD Biosciences), according to the manufacturer’s protocol.

### 2.4 Measurement of plasma cytokines and chemokines

Plasma cytokines and chemokines were quantified using the Beckton-Dickinson Cytometric Bead Array (CBA) Flex Set kits according to the manufacturer’s protocol. The following cytokines and chemokines were quantified: Transforming growth factor-β (TGF-β), Interleukin 5 (IL-5), Interferon-inducible protein 10 (IP-10), Vascular endothelial growth factor (VEGF), Interleukin 6 (IL-6), Tumor Necrosis Factor (TNF), and Interleukin 10 (IL-10). Data was quantified on a BD Accuri C6 Plus cytometer at 448nM and 640nM excitation wave lengths (BD Biosciences, San Jose, CA) and analyzed with FlowJo version 10 software (TreeStar, Ashland, OR).

### 2.5 KSHV neutralizing antibodies

The challenge virus, KSHV (rKSHV.219) encoding the green fluorescent protein (GFP) gene under control of the cellular EF1α promoter, was generated by stimulating latently infected Vero.219 cells, as previously described [13, 16, 17]. Flow cytometry was used to titrate the rKSHV.219 stock to determine the amount required to achieve 50% infectivity on the human embryonic kidney cell line (293T cells). A 1:50 dilution of heat-inactivated plasma was incubated with rKSHV.219 virus, then the virus-plasma mixture was used to infect 293T cells. All assays were carried out in triplicate and infection was quantified by flow cytometry for the number of cells expressing GFP. A sample was considered to be neutralizing antibody positive if it inhibited at least 50% of the infectivity.

### 2.6 Statistical analysis

Baseline characteristics were analyzed using descriptive statistics. The Kruskal-Wallis test was used to compare cytokine/chemokine differences between groups. Comparison of paired data within groups was done using the Wilcoxon matched-pairs signed-rank test. Testing for correlation of continuous variables was done using the Spearman rank correlation. All statistical tests were two-sided, p values <0.05 were considered significant. Stata version 15 (StataCorp LLC, USA) was used for all statistical analyses.

## 3. Results

### 3.1 Baseline characteristics of study participants

We successfully recruited 27 eligible study participants. 5 of the recruited participants were lost to follow up after the baseline assessment, 1 participant was commenced on cytotoxic chemotherapy at another health facility and hence was no longer eligible for follow up, 13 participants had worsening KS within 6 months of ART initiation, and 4 participants had stable disease or regression of KS lesions after at least 6 months of follow up. Among the participants that worsened on ART, only those that were on ART for at least 2 months before worsening of KS were analyzed. We therefore analyzed 11 EpKS patients who had early-stage KS disease and recently initiated ART, 7 of these had EpKS disease progression within 6 months of initiation of ART and follow up while 4 had stable disease or KS tumor regression for at least 6 months. Table 1 highlights the baseline characteristics of the analyzed study participants by treatment outcome.

**Table 1:** Baseline characteristics.

	Poor Responders N = 7	Responders N = 4
Median Age in Years [IQR]	43 [31–55]	38.5 [31–45.5]
Percentage of Males	43	50
Median Time Since HIV Diagnosis in Days [IQR]	7 [2–21]	22 [9–106]
Median Duration on ART in Days [IQR]	2 [1–7]	0 [0–0]
Median Time Since First KS Lesion Noticed in Months [IQR]	3 [3–6]	5 [3–7]

IQR, Interquartile range.

### 3.2 HIV disease parameters

Among the poor responders, the median HIV plasma viral load at baseline was 45,610 copies/ml [IQR = 6,477–691,296] while at determination of ART response (DAR) the median HIV viral load had dropped to 82 copies/ml [IQR = 0–2664]. Responders had a baseline median HIV viral load of 577,016 copies/ml [IQR = 401,840–1,092,995] which dropped to 353 copies/ml [IQR = 15–1679] at DAR. There was no statistically significant difference in HIV viral load at baseline or at DAR between the responders and poor responders. There was a statistically significant decrease in HIV viral load from baseline levels at DAR among the poor responders (p = 0.018), whereas the drop in HIV viral load among the responders was not statistically significant (p = 0.11).

The median CD4 count for the poor responders was 166 cells/μl [IQR = 127–295] at baseline, and increased to 195 cells/μl [IQR = 131–339] at DAR. The median CD4 count for responders was 135 cells/μl [IQR = 68–211] at baseline and was 135 cells/μl [IQR = 101–206] at DAR. There was no statistically significant difference in CD4 counts at baseline and at DAR between responders and poor responders. In addition, CD4 count comparisons between baseline and DAR in both responders and poor responders were not statistically significant.

### 3.3 Comparison of plasma cytokines between responders and poor responders

**Fig 1:**
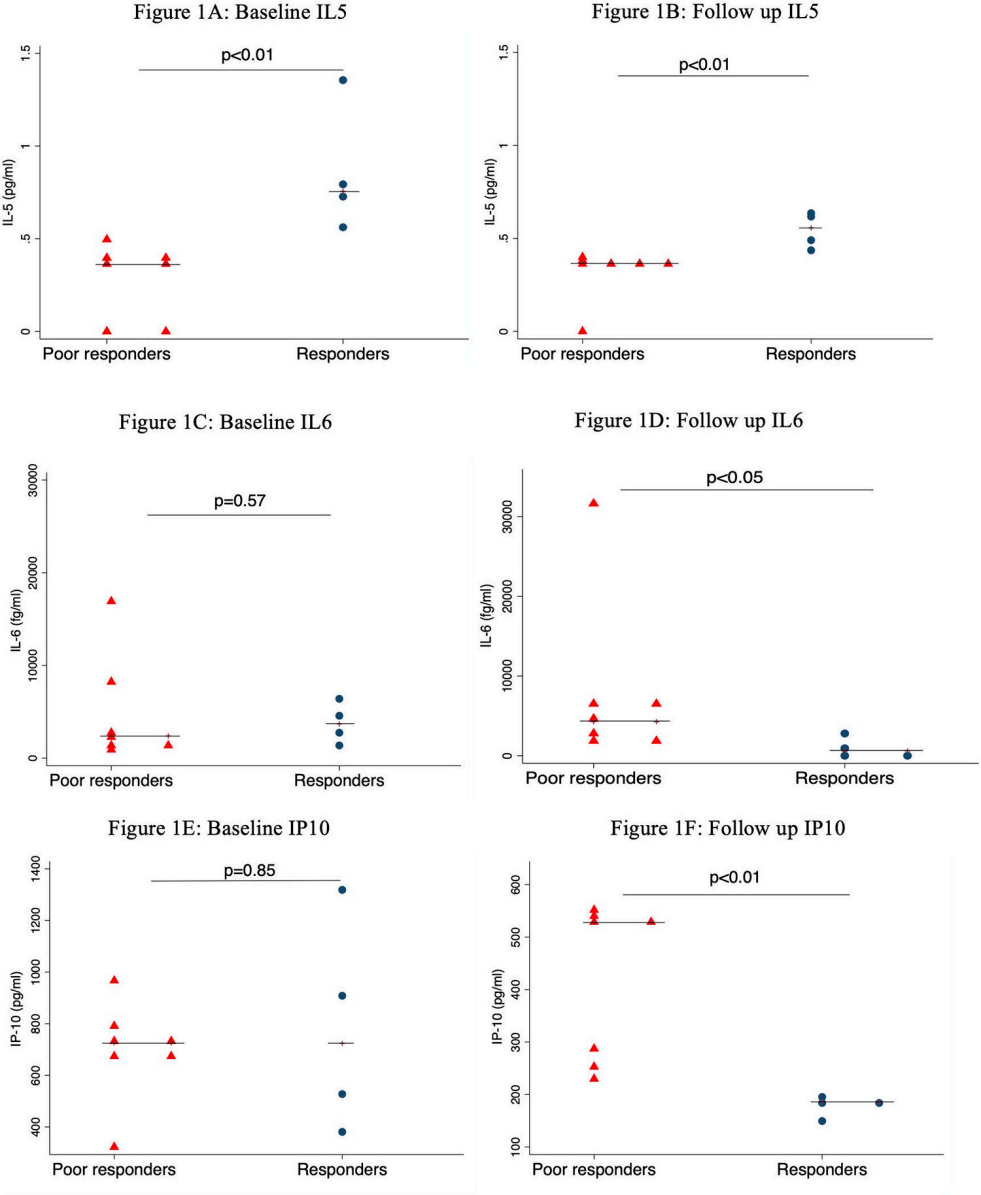
Cytokine levels in responders and poor responders. Bars represent median values.

We quantified the plasma levels of TGF-β, IL-5, IP-10, VEGF, IL-6, TNF, and IL-10 in responders and poor-responders at baseline and at DAR. These cytokines and chemokines have previously been associated with regulation of cell growth and survival, humoral immune response, chemotaxis, tumor proliferation, and enhancing and/or suppressing cancer-associated inflammation [18–22]. There was no statistically significant difference between the two groups at baseline or at DAR in the plasma levels of VEGF, TGF-β, TNF, and IL-10. However, median IL-5 levels were significantly higher in responders than poor responders at baseline (0.76pg/ml vs. 0.37pg/ml; p<0.01) [Fig 1A], and remained higher at DAR (0.56pg/ml vs 0.37pg/ml; p<0.01) [Fig 1B]. There was no significant difference in median IL-6 levels between responders and poor responders at baseline (3688fg/ml vs. 2390fg/ml; p = 0.57) [Fig 1C]; however, at the time of DAR, median IL-6 levels were 7-fold lower in the responders than in the poor responders (600fg/ml vs. 4272fg/ml; p<0.05) [Fig 1D].

The median plasma level of the chemokine CXCL10 (IP-10) was not significantly differential at baseline between responders and poor responders (724pg/ml vs. 726pg/ml; p = 0.85) [Fig 1E]. However, at DAR, the level of IP-10 was significantly lower in responders than poor responders (187pg/ml vs. 528pg/ml; p<0.01) [Fig 1F].

### 3.4 Comparison of KSHV neutralizing antibodies between responders and poor responders

KS patients have much higher neutralizing antibody (nAb) responses than KSHV-infected, but asymptomatic individuals with HIV-1 co-infection [13, 16]. However, whether such responses increase, decrease, or remain unchanged, over the course of ART treatment of early KS is unknown. Moreover, whether the magnitude of the baseline nAb response correlates with eventual ART treatment outcome has not been explored. At baseline, both responders and poor responders were able to neutralize >50% of the challenge virus. Neutralization trended higher in responders than in poor responders; however, this difference was not statistically significant (85% vs. 67%; p = 0.20). At DAR, the magnitude of the KSHV nAb response in responders was also indistinguishable from that in poor responders (66% vs. 70%; p = 1.0). There was a non-significant decline in KSHV nAb responses from baseline to DAR among responders, whereas the nAb responses trended upward over treatment among the poor responders. Neither differential was statistically significant (p = 0.29 and p = 0.75 respectively). No correlation was detected between the levels of antibody-associated cytokine IL-5, and nAb levels at baseline in responders (⍴ = -0.5; p = 0.67) or poor responders (⍴ = 0.46; p = 0.35). At DAR, there was no correlation between IL-5 levels and nAb levels among the poor responders (⍴ = -0.54; p = 0.27), whereas the correlation could not be determined among the responders. Table 2 highlights a compilation of HIV viral loads, CD4 counts, cytokines/chemokines, and KSHV nAb results.

**Table 2:** HIV viral loads, CD4 counts, cytokines/chemokines, and KSHV nAb results.

	Poor responders			Responders				
	Baseline	DAR	*p value*^#^	Baseline	DAR	*p value*^#^	p value*	p value**
**HIV viral load (copies/ml)**	45,610	82	**0.02**	577,016	353	0.11	0.21	1.00
**CD4 Count (cells/μl)**	166	195	0.17	135	135	0.72	0.39	0.29
**IL-5 (pg/ml)**	0.37	0.37	0.87	0.76	0.56	0.07	**0.01**	**0.01**
**IL-6 (fg/ml)**	2390	4272	0.18	3688	600	0.07	0.57	**0.04**
**CXCL-10 (pg/ml)**	726	528	**0.02**	724	187	0.07	0.85	**0.01**
**KSHV nAb (%)**	67	66	0.75	85	70	0.29	0.20	1.00

DAR, Determination of ART response;

^#^Comparison between baseline and DAR values;

*, Comparison between baseline values;

**, Comparison between DAR values.

## 4. Discussion

HIV-induced immune suppression and dysregulation are major predisposing factors for the development of EpKS. However, it is not entirely clear what HIV does and to what extent. Therefore, treatment with ART is essential in the management and long-term control of KS. ART-induced immune reconstitution reverses KS progression [23]. However, in a significant proportion of individuals, ART seems to exacerbate the disease [24]. Changes in immunological and/or HIV and KSHV virological factors likely increase or decrease the likelihood of ART-treated KS regression or progression. In this study, we quantified several immunological and virological factors and their potential association with response to ART treatment of early-diagnosed EpKS.

We observed a decrease in HIV viral loads in both responders and poor responders to viral suppression levels of less than 1000copies/ml. Previous studies have reported that ART has no direct anti-KSHV or anti-KS activity [25]. In addition, there is abundant evidence of KS development in HIV patients on ART who have high CD4 counts and low viral loads [26, 27]. Consistent with the concept, in our study, HIV viral load was not differential between the responders and poor responders at baseline or follow up. In addition, CD4 counts were also not significantly different at baseline and follow up between responders and poor-responders. This suggests that ART reduction of HIV replication is decoupled from KS control even though uncontrolled HIV-1 replication clearly leads to enhanced risk for KS development.

Consistent with previous reports of KSHV nAb primarily in KS symptomatic subjects [13, 16], both responders and poor responders demonstrated high KSHV nAb. However, due to the small sample size, the observation that nAb was higher among responders than poor responders was not statistically significant. In concert with previous reports, the lack of correlation of baseline nAb level, or changes in nAb, with treatment, suggests that KSHV nAb is not a correlate of protection. The data further suggest that nAb is unlikely to be a marker for KS disease progression or control in the context of ART.

We did, however, observe differences in IL-5, IL-6 and IP-10 between responders and poor responders. IL-5 is a Th2 cytokine that induces eosinophilia and also promotes immunoglobulin secretion by B cells [21]. IL-5 levels have been reported to increase following KSHV infection and in KSHV-associated conditions [28, 29]. We observed higher IL-5 levels at baseline and follow up among the responders compared to the poor responders. It was therefore anticipated that anti-KSHV nAb would be significantly higher among responders than poor responders. Yet, IL-5 levels did not correlate with levels of anti-KSHV nAb, suggesting that IL-5 expression may not be a good marker of humoral control of KSHV infection. The high IL-5 levels, may reflect the previously described effect of parasitic infestations on initiation and progression of KSHV pathogenesis [30, 31]. Nevertheless, our study findings suggest that high IL-5 levels at baseline may be a good prognostic marker for ART-treated EpKS perhaps indicative of a more favorable outcome of Th2 versus Th1 skewing of responses.

We also observed higher plasma IL-6 levels in individuals who were undergoing KS disease progression compared to those who underwent response. IL-6 is a pleiotropic cytokine known to stimulate the proliferation of KSHV-infected cells [18, 32], and also promotes humoral responses by driving B cell maturation [33, 34]. Interestingly, KSHV also produces a viral homolog of IL-6 (vIL-6) which upregulates expression of carcinoembryonic antigen-related cell adhesion molecule 1 (CEACAM1), a protein that has been implicated in angiogenesis, endothelial cell migration, and vascular remodeling [35]. Both IL-6 and vIL-6 have been associated with the proliferation of KSHV-infected tumors [36, 37]. Furthermore, detectable plasma KSHV viral load has been found to be associated with elevated plasma IL-6 levels in KS patients [38]. Our findings are consistent with previous reports on the role of IL-6 in promoting the proliferation of KSHV tumors. In turn, it is possible that elevated IL-6 also contributes to the detection of nAb in KS disease, even though such Ab responses are non-protective.

IP-10 is a chemoattractant for recruiting leukocytes to involved tissues, and thereby intensifies inflammation that results in tissue damage [39, 40]. It is also closely associated with HIV infection, systemic inflammation, and cellular activation [19]. In cancer, IP-10 has been shown to both inhibit and promote tumor formation and/or metastasis [20]. The effect of IP-10 on a tumor largely depends on the type of CXCR3 receptor expressed by that tumor. IP-10 has been found to inhibit CNS tumors and melanoma, whereas it has been observed to promote breast cancer, some lymphomas, colon cancer, and basal cell carcinoma [41–46]. The effect of IP-10 on KS tumors is largely unknown. We observed a significant decrease in plasma IP-10 levels among the responders whereas the poor responders experienced no change from baseline, suggesting a potential tumor promoting effect of IP-10 in KS. This is consistent with previous reports on upregulation of IP-10 expression in KS tumors [47].

## 5. Study limitations

The major limitation of this study was the low number of complete responders. A larger sample size with increased numbers of both responders and poor responders, and a longer follow up period would be required in future.

## 6. Conclusion

High plasma IL-5 is a potential marker of good prognosis in ART-treated EpKS, whereas high plasma IL-6 and IP-10 levels are prognostic markers for potentially poor ART-treatment outcomes.
